# Climate Change and Infectious Disease: Is the Future Here?

**DOI:** 10.1289/ehp.119-a394

**Published:** 2011-09-01

**Authors:** Catherine M. Cooney

**Affiliations:** Catherine M. Cooney, a science writer based in Washington, DC, has written for *Environmental Science & Technology* and *Chemical Watch*.


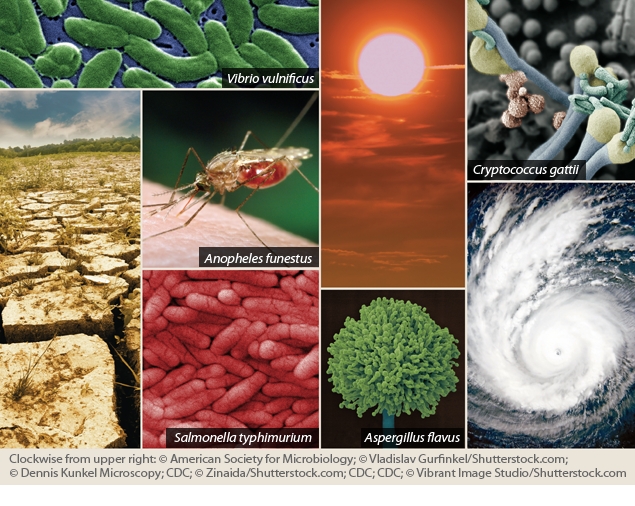
After kayaking on Vancouver Island’s eastern shore, a 45-year-old woman’s headaches and night sweats were little indication that she had been exposed to a rare and dangerous fungus while on the water. Her doctors, mystified as to the cause of the symptoms, didn’t recognize that the infection was *Cryptococcus gattii—*a species of pathogenic yeast—until shortly before her death in 2002.^1^

Most cases of *C. gattii* have been reported in the warmer climates of Australia, Asia, Africa, and Southern California. But at least two strains of the fungus are now affecting humans, pets, and wild animals in the U.S. Pacific Northwest.^2^ From January 2004 through July 2010, a total of 15 people died from *C. gattii* infection, according to the Centers for Disease Control and Prevention (CDC), and 60 human cases of the illness were reported in Oregon, Washington, California, and Idaho.^3^ Despite the alarming sound of the disease, public health officials in Oregon have urged residents not to consider the spread of *C. gattii* as a health emergency—although the fungus is present in the wild, few people have become seriously ill with this infection, and even fewer have died, says Oregon state epidemiologist Katrina Hedberg.

Nevertheless, the mere presence of this foreign species so far from its home raises questions. Reports suggest the fungus may have been exported from its native habitat on commercially valuable trees such as eucalyptus and ornamental *Ficus* species.^4^ “We don’t know exactly why the [Pacific Northwest] outbreak emerged,” says Edmond Byrnes, a postdoctoral fellow at the Johns Hopkins University School of Medicine who has studied the fungus.^5^ “One of the more current hypotheses is that climate change is one of the factors that should be considered.” Byrnes explains that *C. gattii* may be able to establish itself in the Pacific Northwest region because of milder winters with daily average temperatures above freezing.

*C. gattii* is one of several pathogens whose spread is hypothesized to be linked to climate change. Like many aspects of climate change, the connection with infectious disease involves controversy. Some scientists argue that improved climate models may give a false impression that climate change is driving a spread in infectious diseases; others point to human activity and other factors as far more important determinants than climate.^6^^,^^7^^,^^8^^,^^9^

But health practitioners know that a myriad of factors affect the spread of any disease, and many of these—including human migration, poverty, water and air quality, land use decisions, ecological change, the strength of the local public health system, and even access to air conditioning—are themselves intertwined with climate change.^10^ Moreover, says Jonathan Patz, a professor of environmental studies and population health sciences at the University of Wisconsin–Madison, “So many infectious diseases are sensitive to climate that if the majority of the climatologists around the world are telling us that climate is changing and will continue to change under the scenario of global warming, then disease incidence will change.” Several scientific studies and reports suggest these proposed future effects of climate change are in fact already occurring.^11^

## Diseases of Interest

Considering climate change and extreme weather events when analyzing the spread of disease is a fairly new idea. In one of the first papers to call attention to the potential connection, published in 1989, author Alexander Leaf listed immune system depression, health care and sanitation deficiencies, pollution, population shifts, malnutrition, vector shifts, and contaminated water supplies as factors that could spur a rise in infectious diseases in a warming climate.^12^ Studies published as part of a series in *The Lancet* in the fall of 1993 first began to link increased cases of infectious disease to longer seasons, hotter temperatures, and increased rainfall.^13^^,^^14^^,^^15^ Studies published since then have coupled aspects of climate change to increased outbreaks of viral illnesses such as West Nile virus (WNV) and dengue fever, and to outbreaks of bacterial illnesses such as cholera and salmonellosis.^10^ In 1996 the Intergovernmental Panel on Climate Change (IPCC) for the first time included a chapter on public health effects in its report.^16^

To make a firmer determination about links between climate change and infectious disease, researchers need high-quality data collected over long periods detailing changes in the numerous factors that go into the spread of disease. But those data haven’t been collected. “That is one reason some of us look at cases where [there are] unseasonable conditions, like in an El Niño where there are unseasonal rain or drought conditions or unusually high temperatures,” Patz says. “We look at that and say, as scientists, okay what did that extreme weather pattern do to disease? If we can study what happens to disease in extreme weather events, it gives us a window into the future [where such extremes are expected to become more common].”

Most public health officials contacted for this article agree that climate change and extreme weather events will move more infectious diseases northward. Warmer winters and high-latitude warming—occurring twice as fast as overall warming^17^—are already contributing to shifts and expansions of vector ranges.^18^ In addition, extreme weather events—occurring with greater frequency and intensity^19^—are often associated with outbreaks of water-, mosquito-, and rodentborne diseases,^20^ says Paul Epstein, associate director of Harvard’s Center for Health and the Global Environment.

But predicting how the interaction of factors will play out is not always straightforward. According to the World Health Organization (WHO) Scientific Working Group, dengue is the most rapidly spreading vectorborne disease in the world, with the average annual number of reported cases increasing by more than 7.5 times between 1970–1979 and 2000–2005.^21^ Malaria, on the other hand, is decreasing in all regions in response to highly targeted control efforts—in 2009 there were an estimated 225 million cases and 781,000 deaths worldwide, down from 233 million cases and 985,000 deaths in 2000.^22^ In the *World Malaria Report 2010* the WHO stated, “A realistic view of what would have happened without control activities . . . cannot be established from the data currently available,” but suggested that, absent control activities, short-term climate variations could be expected to affect disease trends.^22^

Lyme disease, spread by ticks carrying the bacterium *Borrelia burgdorferi*, has been expanding for decades in the United States, but it is difficult to know how much of that expansion is a result of infected ticks expanding their geographic range versus growing awareness and better detection of the disease. It is clearly both, says Richard S. Ostfeld, a disease ecologist with the Carey Institute of Ecosystem Studies in Millbrook, New York. “We know [Lyme disease] has spread into the mid-Atlantic states, into Maryland and Virginia and north into New Jersey and New York and southern Canada,” he says. “It is possible that the spread northward is influenced by a warming climate, but that wouldn’t explain the spread southward. We just don’t know what the other factors are.”

Other diseases are predicted to become more prevalent as a result of climate-related changes in water and food sources. Water contamination from flooding can cause shortages of clean water that lead to the spread of diarrheal diseases such as cholera as well as enteric diseases such as typhoid.^23^ Not having enough water for cleaning and bathing can cause infections such as scabies^24^ and trachoma,^25^ whereas drinking too little water can lead to harmful infections of the bladder and kidneys.^26^ Warm temperatures and rainfall have also been tied to the spread of foodborne contaminants. For example, contamination of crops with aflatoxins—potent mycotoxins produced by *Aspergillus flavus* fungi that can cause developmental and immune system suppression, cancer, and death—is linked both to increased rainfall and to drought.^27^

One thing that seems fairly clear is that not all areas will see uniform shifts in infectious diseases; these, like other climate-related changes, will be highly dependent on local factors.^10^ “In some areas [in the United States] we expect to see a disease increase, and in others areas we expect to see a decrease. How climate change will affect that is really a wildcard at this time,” says Ben Beard, associate director for climate change at the CDC’s National Center for Emerging and Zoonotic Infectious Diseases.

## A Wakeup Call

The U.S. public health system was tested a decade ago when WNV first appeared in this country. WNV is a member of the genus *Flavivirus* (relatives include the viruses responsible for yellow fever and malaria).^28^ The virus spread rapidly upon its 1999 emergence in the United States and is now endemic in several states. In 2010 it caused a total of 1,021 human disease cases and 57 deaths; in years prior, the number of cases reached as high as 9,862 and the number of deaths reached 264.^29^ The virus has been shown to develop more rapidly during hot weather, with epidemic conditions observed during just-above-average temperatures in California and other temperate regions of the United States.^30^

The emergence of WNV was a wakeup call for many public health specialists who hadn’t seen such outbreaks before. “Before West Nile virus was introduced in the United States and then took off, as a medical entomologist I would have said something like this would not happen in the United States . . . but it did happen,” says Daniel Strickman, national program leader for veterinary, medical, and urban entomology with the Agricultural Research Service.

As many as 38 states have developed or are developing climate change action plans, according to the Pew Center on Global Climate Change.^31^ But planning for outbreaks of new or previously eradicated diseases is typically not at the top of a state’s climate adaptation plan. “When you look at where the rubber hits the road and what is going on at the state level to respond to or prepare for health effects from climate change, it always boils down to heat waves,” says Art DeGaetano, a professor in the Department of Earth and Atmospheric Sciences at Cornell University.

Paul Jarris, executive director of the Association of State and Territorial Health Officials, explains that state infectious disease teams and epidemiologists do look at reemerging and new diseases, “but not necessarily through the climate adaptation plan because these [diseases] are considered secondary threats to heat [and] flooding.” Jarris says he thinks states have done well with limited resources when confronted with unusual disease outbreaks, responding with supportive care, vector control programs, and public education to treat and reduce the spread of disease. State and local emergency preparedness and response personnel have been called in to respond to extreme weather situations, and sanitation and water inspectors have helped identify the effects of excessive rainfall, such as stormwater runoff and well contamination that fouls drinking water.

Some states do have surveillance and monitoring programs to track types and locations of mosquitoes, some analyze the insects for emerging viruses, and many track the incidence of mosquito- and tickborne diseases. For example, mosquito control agencies in California test insects for WNV and other, potentially newly introduced arboviruses, and for newly introduced mosquito species that may be capable of transmitting pathogens for diseases not currently endemic (such as dengue fever and chikungunya^32^), says Gilberto Chavez, deputy director of the Center for Infectious Diseases at the California Department of Public Health. Despite these efforts—and despite the fact that California has the most aggressive climate change mitigation law in the country—there are no state-level adaptation discussions under way specifically concerning climate change and infectious disease, Chavez says.

This situation is not at all unusual, according to George Luber, associate director for climate change at the CDC. “When we go into states and talk to medical officials, public health officials, and across community sectors to urban planners or water managers, they are frankly surprised to hear that we are seeing that climate has an impact on health right now and that it will become more serious in the future,” he says.

The struggling economy and politics make it tough for public health officials to move proactively. “There is a particular challenge when you are dealing with climate change because of the political interpretation of the science,” Jarris says. “If you go before a state legislature asking for funding . . . you can run into a political buzzsaw. It is very difficult to get funding for new initiatives in the current fiscal environment.”

Moreover, says Emily C. Zielinski-Gutierrez, a behavioral scientist with the CDC Division of Vector-Borne Diseases, “In this time of economic challenge, some states are scaling back their public health resources, which is a significant concern because those states won’t be prepared when something does break out.”

## Collaborating for the Future

In September 2011 the CDC—which is leading the federal government’s program to assist states and localities in addressing health issues related to climate change—plans to add climate indicators on its existing National Environmental Public Health Tracking Network.^33^ This searchable product of the CDC’s National Environmental Public Health Tracking Program provides users with integrated health, exposure, and hazard information and data from a variety of national, state, and city sources. The indicators provide consistent and standardized methods for comparing public health surveillance and environmental monitoring data across multiple states. They will help public health specialists begin to detect any emerging patterns that connect disease outbreaks with climate events, Luber says.

Disentangling the complex relationship between climate change and infectious diseases will require collaborations involving epidemiologists, disease ecologists, climatologists, modelers, geographic information specialists, sociologists, economists, and policy experts, says Rita Colwell, Distinguished University Professor at the University of Maryland at College Park and at Johns Hopkins University Bloomberg School of Public Health. In small steps, this type of collaboration is beginning to take shape.

For example, last spring the Johns Hopkins University Applied Physics Laboratory (APL) launched GAIA (Global Assimilation of Information for Action), which features online symposia designed to let climate researchers and public health practitioners share their expertise and data. Larry J. Paxton, staff scientist and head of the APL Atmospheric and Ionospheric Remote Sensing Group, says he initiated GAIA in order to apply the principles of systems engineering—an interdisciplinary approach to managing each aspect of a large engineering project over its lifetime—to the impacts of climate change being seen around the world. Like Colwell, Paxton sees climate change as a diverse issue that is best served by interdisciplinary, collaborative efforts.

In another step, two postdoctoral students just completed their first year of a CDC-sponsored program to cross-train recent public health graduates. The program’s goal is “to get some formal cross-training between public health professionals and climatologists, and hopefully begin to crack some new ground,” Luber says. The students have finished up one year at the National Center for Atmospheric Research in Boulder, Colorado, and will spend the next at the CDC studying climate change–related health effects; one student will focus on vectorborne diseases, Luber says.

Many public and clinical health professionals aren’t sure where to find or how to use local and state climate data. In order to begin a conversation between health practioners, climatologists, and meteorologists—so each group can become aware of the knowledge, skills, and needs of the other—in July 2011 the CDC and other agencies cosponsored a Colloquium on Climate and Health on vectorborne diseases.^34^ The weeklong workshop gave state and local public health officials hands-on experience with climate modeling and meteorology practices, as well as talks by vectorborne disease specialists. Over the past 2 years, the Harvard Center for Health and the Global Environment has held state-level courses on health and climate change in conjunction with the American Medical Association.

The CDC’s Climate-Ready States and Cities Initiative recently awarded grants to public health departments in eight states and two cities to help them build capacity for climate change–related health effects.^35^ The grants will fund health impact assessments and capacity building to prepare for vector-, food-, and waterborne diseases.

Meanwhile, research on the connections between climate and infectious disease continues. In one new initiative, Matthew Thomas, a professor of entomology at Penn State’s College of Agricultural Sciences, is leading a group of scientists who are studying how temperature influences the spread of dengue and malaria and how that can determine disease risk. Findings from this project should provide more clues about the spread of other vectorborne illnesses, which in turn can help health experts develop disease prevention and control tactics, Thomas says.

At the same time, Thomas and many other researchers stress that it’s time to push ahead in science and try to better understand the many factors that go into a disease outbreak. He says, “We need to move beyond these extreme cases of asking if climate change does or does not affect the spread of disease.” Instead, he says, researchers should embrace the many and complex factors—including but not limited to climate—that work together to initiate the spread of infectious disease.
